# Stories of change in nutrition: lessons from a new generation of studies from Africa, Asia and Europe

**DOI:** 10.1007/s12571-022-01314-8

**Published:** 2022-09-19

**Authors:** Nicholas Nisbett, Jody Harris, Derek Headey, Mara van den Bold, Stuart Gillespie, Noora-Lisa Aberman, Olutayo Adeyemi, Richmond Aryeetey, Rasmi Avula, Elodie Becquey, Scott Drimie, Elyse Iruhiriye, Leah Salm, Zuzanna Turowska

**Affiliations:** 1 Institute of Development Studies, University of Sussex, Sussex, UK; 2 World Vegetable Centre, Bangkok, Thailand; 3 International Food Policy Research Institute (IFPRI), Washington, D.C USA; 4 Clark University, Worcester, USA; 5 Global Alliance for Improved Nutrition, Washington, D.C USA; 6 Department of Human Nutrition and Dietetics, University of Ibadan, Ibadan, Nigeria; 7 School of Public Health, University of Ghana, Accra, Ghana; 8 Department of Global Health, Faculty of Health and Medicine Sciences, Stellenbosch University, Stellenbosch, South Africa; 9 Johns Hopkins Bloomberg School of Public Health and University of South Carolina Arnold School of Public Health, Baltimore MD and Columbia, SC USA; 10 Independent Consultant for IFPRI, Dakar, Senegal

**Keywords:** Malnutrition, Obesity, Policy, Commitment, Coherence, Equity

## Abstract

How does nutrition improve? We need to understand better what drives both positive and negative change in different contexts, and what more can be done to reduce malnutrition. Since 2015, the Stories of Change in Nutrition studies have analysed and documented experiences in many different African and Asian countries, to foster empirically-grounded experiential learning across contexts. This article provides an overview of findings from 14 studies undertaken in nine countries in South Asia, sub-Saharan Africa, and Europe between 2017 and 2021. The studies used a combination of methods, including regression-decomposition analyses of national datasets to assess determinants of nutritional change; policy process and food environment analyses; and community-level research assessing attitudes to change. This article takes a narrative synthesis approach to identify key themes across the studies, paying particular attention to multisectoral determinants, changes in the food environment, the role of structural factors (including longstanding social inequities), and changes in political commitment, cross-sectoral coherence and capacity. Given the inherent multisectoral nature of nutrition, many countries are experimenting with different models of ensuring coherence across sectors that are captured in this body of work. The relative immaturity of the policy sector in dealing with issues such as obesity and overweight, and associated influences in the wider food environment, adds a further challenge. To address these interrelated issues, policy must simultaneously tackle nutrition’s upstream (social/economic/equity) and downstream (health and dietary) determinants. Studies synthesised here provide empirically-driven inspiration for action.

## Introduction and background

Malnutrition has many faces, from child stunting to adult obesity to widespread micronutrient deficiencies. Data are accumulating that enable estimating prevalence of these public health outcomes for different populations and locations (Development Initiatives, 2020). There is, however, a growing need to understand what is driving both positive and negative change, and therefore what more can be practically done to reduce malnutrition.

Change in malnutrition outcomes is driven by changes in the known determinants of malnutrition. Following the UNICEF conceptual framework, and its adaptation for the Lancet Series on Maternal and Child Nutrition, these include determinants at *underlying* (food, health and available care) as well as *basic* levels (poverty, education, access to services) (Black et al., 2008, 2013; UNICEF, 1990). What shapes these determinants in different contexts is influenced by the actors (institutional or individual, public or private) involved in the design of public policies and their actual implementation (Gillespie et al., 2013), societal norms and values shaping who has access to what (Nisbett et al., 2021), and structural factors such as economic arrangements (e.g., liberalization policies, trade agreements) and social inequities (e.g., along axes of race or sex), including those shaped by colonial legacies (Nisbett et al., 2022).

While the UNICEF/Lancet conceptualisation of malnutrition represents a consensus within the nutrition community, the mix of determinants will vary in importance across contexts. In practice, more experiential learning is needed to understand the processes through which nutrition changes, and to support policymakers, implementers, and advocates to decide on practical actions. Since 2015, the Stories of Change in Nutrition studies have analysed and documented these experiences in different African and Asian countries, with a view to providing empirically grounded experiential learning across contexts (Gillespie & van den Bold, 2017). This work has (1) quantified the contribution of different determinants to changes in child stunting; (2) analysed how the public policy environment has shaped action on malnutrition; and (3) used a variety of qualitative interview methods to capture the lived experiences of different communities through the course of these policy and outcome changes.

Building on this earlier work, this latest set of studies (2017—2021) sought to: (1) broaden analyses of outcomes from stunting to malnutrition in all its forms (including overweight/obesity and micronutrient deficiencies); (2) understand the specific combinations of underlying and basic determinants in different contexts, and (3) understand how the policy environment shapes these determinants to enable or constrain effective action for different groups, using an equity lens. This article is intended to provide an overview and synthesis of the findings from 14 studies undertaken for the most recent phase of the Stories of Change initiative, published between 2019 and 2022. Here, we outline commonalities and differences across studies, and we consider the approaches that seem to yield success in different contexts to reduce all forms of malnutrition for everyone, in all the studies conducted.

In Africa, studies took place in Ghana (Aberman et al., 2022; Aryeetey et al., 2021), Nigeria (Adeyemi et al., 2022; Adeyemi et al., Forthcoming), Burkina Faso (Becquey et al., 2022; Turowska et al., Forthcoming), Rwanda (Iruhiriye et al., 2022), Zambia (Harris et al., 2019) and South Africa (Drimie et al., Forthcoming). In South-East and South Asia, studies took place in Vietnam (Harris et al., 2020, 2021, 2022) and across four states of India (Odisha, Tamil Nadu, Gujarat & Chhattisgarh) (Avula et al., 2022; Avula et al., Forthcoming). In Europe, a city-level study took place in Brighton and Hove in the United Kingdom (Salm et al., Forthcoming). In total, the 14 studies summarised here cover nine countries, as four studies took place in India and three took place in Vietnam.

## Methods

The 14 Stories of Change studies^1^ all have the objective of understanding what drives change in different forms of malnutrition in different contexts. Studies used a combination of methods and frameworks to achieve this objective, triangulating between data and evidence and the perspectives of nutrition actors to tell this ‘story of change in nutrition’. All studies offer research questions that are specific to the country and the story in question. In synthesising the studies, therefore, we aim to produce a new set of analytical themes. This leads us to articulate further (post-hoc) questions which we felt were informed by the collection of studies. These new questions emerging from our synthesis constitute the sections of the present article.

Studies aiming to understand the determinants of stunting reduction (and anaemia in the case of Ghana) in different contexts (Adeyemi et al., 2022; Aryeetey et al., 2021; Avula et al., 2022; Becquey et al., 2022; Harris et al., 2021; Iruhiriye et al., 2022) built on previous work (Headey et al., 2016, 2017) and applied regression-decomposition analysis on large national datasets over multiple years (Table 1). Many studies used and adapted the 1990 UNICEF framework on the causes of child and maternal malnutrition in the identification of underlying and basic determinants of change in these outcomes. This UNICEF framework was also used to identify independent variables to involve in multiple regression and regression decomposition analyses. Two studies (Adeyemi et al., 2022; Harris et al., 2021) disaggregated these analyses (one by geography, the other by ethnic group) in order to understand inequality in outcomes for different population groups.

**Table 1:** Outcomes and time periods studied

**Stories of Change Study**	**Study Period**					
	** < 2000**	**2000–2004**	**2005–2009**		**2010–2014**	**2015–2019**
**Chhattisgarh (India)**			**2006**			**2016**
Stunting (%) (< 5 years)			53			38
**Gujarat (India)**						
Stunting (%) (< 5 years)			51			38
**Odisha (India)**						
Stunting (%) (< 5 years)			45			34
**Tamil Nadu (India)**						
Stunting (%) (< 5 years)			32			27
**South Africa**	**1999**	**2003**	**2005**		**2012**	**2016**
Stunting (%) (1–3 years: 1999, 2005, 2012; < 5 years, other years)	25.5	27.4	23.4		26.5	27
Wasting (%) (1–9 years: 1999, 2005; < 5 years, other))	3.7	5.2	4.5		2.9	2.5
Adult Overweight (%)	12.4		10.6			13.3
Adult Obesity (%)	6.6		4.8			not reported
Girls Overweight (%)					16.5	
Girls Obesity (%)					7.1	
**Ghana**		**2003**	**2008**		**2014**	**2017/2018**
Stunting (%) (< 5 years)		29.9	28		18.8	18
Wasting (%) (< 5 years)		7.1	8.5		4.7	7
Anaemia, women (15–49 years)		44.7	58.7		42	
Female (15–49 yr) Overweight (%)		17.2	20.7		24.8	
Female (15-49 yr) Obesity (%)		8.1	9.3		15.3	
Anaemia, Children (< 5 years) (%)		76.1	77.9		65.7	
**Burkina Faso**	**1999**	**2003**			**2010**	
Stunting (%) (< 5 years)	38.6	38.7			35	
Wasting (%) (< 5 years)	13.2	18.6			15.5	
Anaemia, women (15–49 years)		53.7			48.8	
Adult Overweight (%)	5.7	9.3			11.2	
Adult Obesity (%)	0.9	2.4			3.1	
Anaemia, Children (< 5 years) (%)		91.5			87.8	
**Nigeria**		**2003**	**2008**		**2013**	**2018**
Stunting (%) (< 5 years)		42.6	40.6		36.8	36.5
Wasting (%) (< 5 years)		11.3	13.9		18.4	6.9
Anaemia, women (15–49 years)						57.8
Child Overweight (< 5 years) (%)		5.7	8.7		3.9	2.3
Female (15–49 yr) Overweight (%)		14.8	16.1		17.2	18.2
Female (15-49 yr) Obesity (%)		5.8	6		7.5	9.9
Anaemia, Children						67.9
**Rwanda**		**2005**			**2010**	**2015**
Stunting (%) (< 5 years)		51			44	38
Wasting (%) (< 5 years)		5			3	2
Anaemia, women (15–49 years)		26			17	19
Adult Overweight (%)						
Female (15–49 yr) Overweight or Obesity(%)		12			14.1	17
Female (15-49 yr) Obesity (%)					2.2	4
Child overweight or obesity (%)					7	8
Anaemia, Children (%)		52			38	37
**City of Brighton & Hove, UK**			**2008–2009**			**2018–2019**
Child Overweight and obesity (at 4–5 years) (%)			22.8			20
Child Overweight and obesity (at 10–11 years) (%)			31.5			25.6
**Vietnam**		**2000**			**2010**	
Stunting (%) (< 5 years)		36.6			18	
Wasting (%) (< 5 years)		5.7			3.7	
Underweight (%) (< 5 years)		33			15.4	
Note on Sources:						

India NFHS 2006, NFHS 2016, South Africa NFCS 1999, SADHS 2003, FNCS-FB 2005, SANHANES 2012, SADHS 2016, Ghana DHS 2003, DHS 2008, DHS 2014, MICS 2017/2018, Burkina Faso DHS 1999, DHS 2003, DHS 2010, Nigeria DHS 2003, DHS 2008, DHS 2013, DHS 2018, Rwanda DHS 2005, DHS 2010, DHS 2015, City of Brighton and Hove, UK NCMP, Vietnam MICS 2000, 2010

Studies aiming to understand broader drivers of food systems and food environments and their links to the nutrition transition (Aberman et al., 2022; Drimie et al., Forthcoming; Harris et al., 2019; Salm et al., Forthcoming) mostly provided descriptive statistics only, because full datasets describing all components of the food system are not available. These articles describe aspects of food systems as they exist now, or with regards to how they have changed over time, and summarise how such changes might influence change in nutrition, via narrative methods.

Studies aiming to assess the public policy environment (Adeyemi et al., Forthcoming; Aryeetey et al., 2021; Avula et al., 2022; Drimie et al., Forthcoming; Harris et al., 2022; Harris et al., 2021; Iruhiriye et al., 2022; Salm et al., Forthcoming; Turowska et al., Forthcoming) drew on multiple policy analysis frameworks and methods. Policy frameworks included the community readiness framework (Plested et al., 2007), the policy space (Grindle & Thomas, 1991), advocacy coalitions (Sabatier, 1988), and the nutrition-enabling environment (Gillespie et al., 2013). The majority of these studies reviewed written policy to understand how different sectors addressed nutrition issues, including the completeness and coherence of the body of written policy for addressing different nutrition issues. Most studies also undertook key-informant interviews with policy actors at national and sometimes state, provincial, or local government levels, to understand how different actors drew on ideas, beliefs, and interests in shaping the policy process. Some studies complemented these with stakeholder mapping to understand how actors interacted in the policy process (Adeyemi et al., Forthcoming; Aryeetey et al., 2021; Turowska et al., Forthcoming).

Four studies (Aberman et al., 2022; Becquey et al., 2022; Iruhiriye et al., 2022; Salm et al., Forthcoming) undertook interviews and/or focus groups at community level, aiming to understand the perceptions, attitudes or practices of different community groups to different nutrition issues and the policy or implementation environment shaping their lives. Other studies report separately on community level findings and are not included in this synthesis.

In this paper, we provide a synthesis of current and emergent themes based on the articles of this journal’s Series “Stories of Change in Nutrition”.

## Study limitations

There are several caveats that are important to consider in the studies reported in the Series “Stories of Change in Nutrition”. First, the decomposition analysis is only based on observable and available data: the absence of variables in the data sets used, whether observable (notably dietary change, hygiene, disease history, and exposure to nutrition-specific interventions) or unobservable (such as community or household level shocks) explains why decomposition analyses sometimes do not describe fully the variation in observed change, such as child stunting or anaemia. Measurement error – including the limited ability for some variables to adequately measure quality of resources (such as water, sanitation or healthcare) – may also reduce explanatory power. Second, as studies used both quantitative and a variety of qualitative methods, we follow a narrative approach to synthesize findings across articles: this is not a formal approach to synthesizing findings (e.g. via coding articles according to pre-set themes), but allows deriving hypotheses from multiple data sources (triangulation). Third, these case-studies differ substantially, which prevents this synthesis to suggest universally applicable solutions. However, the number of cases enables framing a broad range of actions and hypotheses.

Nevertheless, this set of studies constitutes one of the most comprehensive set of action-oriented research on the multiple burdens of malnutrition in the Global South. All these studies entail the assessment of both basic and underlying determinants within given public policy environments. Collectively, these studies represent a basis for national planning and research.

## Synthesis of findings

We grouped findings into four themes reflecting the following questions:

- What are the main contributions to changes in nutritional status from different government sectors known to affect malnutrition? Which are the policy and operational sectors that tend to be overlooked by nutrition actors?
- What changes have occurred in the food environment, and what is the strength of the policy environment with regard to tackling the growing prevalence of overweight and obesity?
- What was the role of structural change and related policies, including wealth distribution and equity, in access to services?
- What were the changes to the policy environment, particularly with regard to political commitment, cross-sectoral coherence and capacity?

### Trends and associations in determinants of change

Table 2 summarises findings from regression-decomposition analyses designed to explore potential determinants of linear growth improvements (stunting reduction or Heigh-for-Age Z score, HAZ, increases) in the different studies. The case studies are an interesting mix, covering two West African countries with modest improvements in linear growth (Burkina Faso and Nigeria) and another with more rapid improvements (Ghana, where stunting fell from 28 to 18% over 2003–14), as well as Rwanda, an East African country that saw a rapid and large reduction in stunting (51 to 38% between 2005 and 2015). In India, four diverse states were examined, including three states with high initial stunting rates but rapid change over 2005–2016 (Gujarat, Chhattisgarh & Odisha), and one with much lower stunting to begin with due to declines in earlier decades (Tamil Nadu). Finally, Vietnam saw stunting prevalence halve in just 10 years, from 37 to 18% from 2000 to 2010, although that study also focused on disparities between minority ethnic groups and the majority of the Vietnamese population.

**Table 2:** Summary of results from regression-decomposition analyses of determinants of changes in child Height for Age Z-Scores (HAZ) or stunting in six studies

**Country & time frame**	**Observed stunting or HAZ change**	**Stunting/HAZ change explained**	**Explanatory variables with large changes over time**	**Main contributors to explained stunting reduction (%)**
Burkina Faso, 1998–2010	HAZ improved from -1.74 to -1.38	48% of HAZ explained	Immunizations (21–76%); asset accumulation increased modestly (1.6 to 2.4 out of 10); open defecation fell from 81% to 68%. Other changes modest.	Immunization coverage (23%), asset accumulation (10%), reduction in open defecation (6.1%), parental education (3.8%), reduced fertility (1.7%), piped water (1.4%), maternal height (1.8%), antenatal care (0.47%).
Ghana, 2003–14	Stunting fell from 28% to 18% nationally, but increased in Northern Region. HAZ increased rapidly	< 50% of stunting explained; 65% of HAZ explained	Bednets 25–81%; asset index (2.6 to 4.1 out of 10); ANC attendance 70–87%; medical facility births (41–69%); improved water 40–79%.	In both the stunting and the HAZ model, insecticide treated bed nets were the main driver (39% in HAZ model), followed by assets (32% in HAZ model), ANC visits (14%) and medical facility births (11%).
Nigeria, 2003–18	Mean HAZ unchanged, but stunting fell from 42.6% to 36.5%, mostly over 2013–18. Significant regional variation	50% of stunting explained	Maternal education (2 years) and paternal education (1.6 years); ANC visits (51–61%); medical facility births (37–46%); small increase in asset index and other variables.	Maternal education (17%); Asset index (14%); medical facility births (6%), paternal education (5%), child illness (4%), low maternal BMI (3%).
Rwanda, 2005–15	Stunting fell from 52% to 38%	123% of stunting explained	ANC visits (17%) & quality (1.3 to 4 out of index of 0–5); Maternal schooling (0.7 years); vaccination coverage (14%); fertility (4.5 to 3.4 children).	ANC quality (59%), medical facility births (18%), asset accumulation (10%); fertility reductions (6%); parental education (4%), health insurance coverage (4%).
Indian states, 2005–2016 Chhattisgarh, Gujarat, Odisha, Tamil Nadu	Stunting rates fell as follows: 53–38% in Chhattisgarh; 51–38% in Gujarat; 54–34% in Odisha; 32–27% in Tamil Nadu	Stunting models: 66% in Chhattisgarh; 60% in Gujarat; 86% in Odisha; 100% in Tamil Nadu	Rapid changes in a wide range of indicators: Living conditions (assets, electricity), age at marriage, women’s education, ANC visits, skilled birth attendance, immunization, sanitation.	Household living conditions (22–47%); Health & nutrition interventions (11–23%); Maternal factors (15–30%); Community factors (7–19%).
Vietnam, 2000–10	Stunting fell from 37 to 18%; HAZ improved from -1.57 to -0.93 Ethnic minorities experienced similar trends, but stunting 14 points more prevalent in 2000 & 17 points more prevalent in 2010	91% of HAZ explained	Rapid changes in a wide range of indicators: asset accumulation, maternal education, health care indicators, water and sanitation	Wealth index (61%), Health & nutrition interventions (16%), Maternal education (12%), fertility (1.6%).

While all the studies used similar regression decomposition methods and often used similar explanatory variables – which are quite standardized in both the DHS and MICS, there were some variations discussed in individual papers. All studies used a relatively similar asset index scaled from 0–10 to measure the important factors of economic status, and all studies covered parental education. All studies also specified water and sanitation, with the latter specifying either improved sanitation or community-level open defecation. Some measure of fertility was included (e.g. number of children in total or under-5), as were several maternal conditions in addition to education, such as BMI, height, age, or age at marriage.

Perhaps unsurprisingly, the models had large differences in their ability to explain observed changes in stunting or HAZ over time. Interestingly, the three West African case studies only explained around half of stunting variation, though the Ghana HAZ model explained 65% of that country’s quite rapid improvement. In contrast, the Rwandan model slightly over-predicted stunting change (16 points in the model compared to 13 points in the surveys), while the Indian state models explained 60–86% in the three higher-stunting states and 100% in Tamil Nadu (which admittedly only saw a 5-point improvement). Finally, despite such rapid improvements, the stunting model performed very well in Vietnam, accounting for 91% of the change. In general, it seems like more rapid improvements in linear growth are easier to explain than smaller improvements, but data quality may also be a factor; the 1998 Burkina Faso DHS did not collect some key indicators (e.g. malaria prevention or treatment measures), and DHS data quality in Nigeria is notably poor, as the authors note.

What about the explanatory factors predicting stunting/HAZ change over time? These, too, are diverse, although a commonality among the more successful countries/states is the contribution of nutrition-related health services such as antenatal care and medical facility births (and immunization coverage in Burkina Faso, though this may proxy access to other health services). A second factor of note is asset accumulation, which appears to not only explain change within the case studies but also between them. Vietnam saw the most rapid growth in household assets (and this explained 61% of stunting reduction over 2000–2010), but Indian states also saw rapid changes in household living conditions (again with predictive power) and rapid reductions in stunting, as did Ghana. Rwanda is something of an exception with ANC quality the largest determinant of stunting reduction, while the more modest improvements in living conditions in Burkina Faso and Nigeria coincide with small improvements in linear growth.

Maternal factors are also invariably statistically associated with lower stunting risks in the regression models, but their ability to explain improvements in linear growth over time typically vary because of large differences in trends. WASH conditions, in contrast, seem to have less robust associations with HAZ/stunting, perhaps because water/toilet types are poor proxies for water/toilet quality and overall hygienic behaviours. Finally, fertility indicators are often statistically significant in regression analyses, but have mostly changed slowly over time in these studies, or were already relatively low to begin with (Rwanda being a partial exception).

What do these analyses tell us about stunting reduction? First, rapid reductions in stunting require commensurately rapid improvements across a wide range of child, maternal and household welfare dimensions, and – at a government and NGO level – a wide range of sectors. Multi-dimensional poverty reduction is critical, including both household assets but also access to health, WASH and education services and infrastructures. Vietnam saw rapid economic growth driven mainly by private investment, but the government still plays a crucial role in health, education and infrastructure. In contrast, the Nigeria case study uses the regression coefficients to conduct a forward-looking decomposition/prediction to assess how much faster improvements in wealth, health, education and WASH – i.e. matching the recent improvements of the best-performing region in Nigeria—could help the country reduce stunting from 37% to 27% over 2018–2015 (as opposed to a business-as-usual improvement of 37% to just 34%). Of course, the enabling conditions for achieving rapid multi-sectoral improvements for nutrition are the subject of more qualitative research, summarized below.

### Key sectoral determinants of change in nutrition

Malnutrition’s multi-causal aetiology remains a key tenet of public health nutrition, epitomised by the UNICEF framework on the determinants of child and maternal malnutrition (UNICEF, 1990), with its health, care and food pathways. The Stories of Change studies allow for a unique national-level and cross-country comparison of how different sectors are contributing to nutritional change. Such findings are important for those working within and outside of nutrition to understand the ways in which different sectors – from health, to education, to sanitation, to efforts to address gender and other social disparities – can contribute to nutritional change. Studying the contributions of various sectors is also important for identifying gaps and untried levers for change, which might exist in particular national situations.

The health sector was the best represented amongst the sectors reviewed, reflecting the centrality of health ministries to nutrition programming in many countries and the contribution of nutrition-sensitive programmes such as access to antenatal care to nutrition outcomes (though we note an intrinsic bias that the DHS data used by most studies will naturally lend support to health outcomes). Within health, improvements in the coverage of maternal and child health services (e.g. antenatal care, institutional delivery, immunisation) contributed the most to improvements in stunting as indicated in the decomposition analyses. Stakeholders attributed strengthening of vertical programming in the health sector through increased capacity and training of health workers to improvements in the delivery of these. Programmes and policies specifically designed to target nutrition mentioned by stakeholders included infant and young child feeding (IYCF) support, access to micronutrient supplementation including iron and folic acid (IFA), and improved forms of management of acute malnutrition. In Rwanda, increased access to health insurance via Rwanda’s national health insurance programme was also an important finding (Iruhiriye et al., 2022).

Other sectoral improvements at household and community level included those driven by growing access to sanitation, or piped water (Becquey et al., 2022; Turowska et al., Forthcoming). In some countries, improvements in this sector also reflected significant government initiatives, such as a National Sanitation Programme (Avula et al., 2022), and a range of national sanitation policies and action plans (Aryeetey et al., 2021). In Nigeria, increased construction of toilets and boreholes and availability of environmental health officers to promote/enforce environmental sanitation was also noted by stakeholders (Adeyemi et al., Forthcoming).

While maternal education was identified in many of the studies as a strong predictor of improvements in child growth, including in Ghana, Nigeria, India and Vietnam, programmatic and policy changes in the education sector are only covered in more detail in the Vietnam and India studies. In Vietnam, primary education is free and has become nearly universal, with 96% of children who start school reaching the end of primary school, though gaps remain for minority ethnicities (Harris et al., 2021). New education policies are starting to target these gaps, though barriers remain in terms of language access and remoteness/accessibility of schools. In India, a combination of national and state level programmes helped improve enrolment in schools, particularly of girls: with state level initiatives in Chhattisgarh, Gujarat and Odisha including incentives such as cash, bicycles or other in-kind incentives, improved sanitation facilities for girls and living accommodation for girls from marginalised communities attending from large distances. Education was also consistently listed in terms of the multisectoral co-ordination undertaken in Rwanda. In Burkina Faso, relatively low levels of educational attainment were associated with improvements in child growth, suggesting much more might be delivered from this sector in future (Becquey et al., 2022).

Other sectors are less examined in the studies. Electrification, for instance, was included in the analysis alongside sanitation in India as a village level factor contributing to stunting, but not in other studies. Village level access to electricity showed marked improvements in Gujarat, Chhattisgarh and Odisha (Avula et al., 2022). Such infrastructure-nutrition links may be a valuable topic of future studies alongside broader health and human development provision.

### Role of food environments

A key difference between this and the earlier generation of Stories of Change studies is the focus in several of the studies on multiple forms of malnutrition (whereas the earlier studies largely focused on stunting and wasting). Studies in Zambia, Vietnam, Ghana, South Africa and the city of Brighton and Hove in the UK considered changing food environments, local perceptions of obesity and overweight and national, subnational and local trends in overweight and obesity. The objective of these studies was to analyse these important trends, but also to provide analysis of related policy environments at local and national levels, by reflecting on the relative maturity of policy in the face of significant disease burdens associated with obesity and overweight in different population groups.

The nutrition transition is a process of demographic and epidemiological change largely understood as the change in diets and nutritional outcomes experienced by people as countries transition from traditional patterns of agriculture and employment to more globalised and processed food markets (Popkin, 1994). Studies of the nutrition transition in Zambia and Vietnam within the series show that undernutrition is falling and obesity and non-communicable diseases are rising (Harris et al., 2019, 2020). Food supply has improved; but nutrient-rich foods in general are relatively more expensive than staples in both countries. Expenditure in Zambia on non-staples has increased—particularly fruits and vegetables, animal source foods, fats and sugars, and processed foods. This increased expenditure is reflected in increased consumption, particularly in wealthier and more urbanized sections of the population, which are also experiencing the greatest burden of overweight, obesity and associated chronic diseases. Processed and snack foods in diets are linked to an increased use of supermarkets and fast-food outlets in Zambia. In Vietnam, fruit consumption in diets has gone up, while vegetable consumption has experienced fluctuations. Meat and milk consumption have increased significantly, and sweets and sweetened beverages have both increased; oils and fat consumption also more than quadrupled between 1985 and 2009. The retail environment in Vietnam is experiencing both continuity and change, with wet markets and (vegetable-rich) food eaten away from the home still common despite a policy of “supermarketization” (Harris et al., 2020) and a trade policy process that does not consider the nutrition and health implications of changes to trade (Harris et al., 2022).

National level and community level responses to the growing prevalence of overweight and obesity are considered in South Africa, Ghana, and the city of Brighton and Hove in the UK. In South Africa, a political economy analysis finds a food environment shaped by powerful food industry and government actors, which has so far failed to engage substantially with a rising rate of diet related non-communicable diseases and persistent levels of undernutrition and hunger (Drimie et al., Forthcoming). In Ghana, interviews at community level in two small but growing small cities (Techiman and Hohoe) are scored as part of a ‘community readiness model’ (Edwards et al., 2000). The analysis (Aberman et al., 2022) explores community self-perceptions of overweight and obesity and associated health risks and readiness to decrease risk within the community. Traditional norms viewing larger body size as a sign of prosperity were frequently blamed for the prevalence of overweight and obesity, while younger and wealthier residents were described as aspiring to smaller body sizes. However, the extent to which healthy diets and lifestyles were seen as related to health (versus globalizing beauty norms) was not always clear. Localized efforts to support healthy lifestyles and raise awareness of health risks were championed by some local leaders. However, applying a health-equity lens (e.g. (Backholer et al., 2014)) reveals the stark absence of “upstream” policy directives, dedicated funding, or structural interventions for healthy diets and lifestyles. Aberman et al. (2022) warn that the assessment of readiness to tackle health risks related to overweight and obesity must acknowledge “the dependence of a given community on the systems, policies, and funding flows from above” (p391). When decoupled from this broader context, community pressure around weight can lead to stigma and shame.

In Brighton and Hove, childhood overweight and obesity prevalence have been bucking the national trend with a declining prevalence, as the national trend has been for prevalence to increase. Interviews with key policy and community actors highlight factors that have created an enabling environment for reducing overweight and obesity (Salm et al., Forthcoming) and a shared ‘whole system’ approach via the city’s acclaimed food strategy. Central to this has been a long-term commitment to ‘good food’, with a highly active network of local food actors, the championing of early years interventions in the city, as well as horizontal partnership across local government, public health and civil society.

### Role of structural change

The extent to which changes in nutrition outcomes can be related to wider economic and societal development is a perennial question for those working in international nutrition. Improvements in nutritional status have always been clearly linked to economic growth and reductions in poverty, but with some elasticity: economic growth is a necessary but, in and of itself, insufficient factor for improving malnutrition (Smith & Haddad, 2015). While studies have shown a clear correlation between economic growth and nutrition improvements, outliers include countries with high growth and low improvements in nutrition and those with low growth and high improvements – a divergence influenced by the relative contribution of nutrition and other sectoral policies (Smith & Haddad, 2015).

In our studies, improvements in household wealth, measured by an asset index constructed from DHS data, were consistently found to explain the greatest, or at least a considerable proportion^2^ of change in measured outcomes for studies that undertook decomposition analysis. Given the household nature of the DHS (i.e., compared to national per capita income estimates), these improvements suggest that the benefits of economic growth *are* being felt at the household level. But the decomposition analysis also indicates that more work is needed to understand socio-economic and other aspects of equity, given that wealthier households (as measured by the asset index), experienced much better outcomes than poorer households. The specific role of social protection schemes (including cash transfers and food-based schemes) is also noted as a potential factor in the analysis of change in the Indian study, where such schemes were vastly expanded from the mid-2000s onwards. The trade policy space analysis in Vietnam found one policy coalition prioritising international trade to enhance economic development (a positive for nutrition) while the nutrition and health implications of specific trade policy provisions were not considered in policy negotiations (a negative for nutrition). This illustrates clear trade-offs even within a single policy process (Harris et al., 2022); further research is required to understand the net implications for different forms of malnutrition and in different population groups.

Several of the studies report on underlying determinants relevant to women’s status, including education (as reported above) and maternal care. In both Ghana and Nigeria, a longer birth interval was also identified as an important factor, suggesting improvements in reproductive health services and/or in decision making capacity of women, alongside general improvements in education and wealth (Adeyemi et al., 2022; Aryeetey et al., 2021). In Rwanda, fewer total births was also associated with positive change (Iruhiriye et al., 2022), as was maternal height in Ghana and Nigeria, which could indicate gender-specific improvements over the life course (Adeyemi et al., 2022; Aryeetey et al., 2021). In India, a combination of national programmes and state specific initiatives were reported as having contributed to an improvement in the status of women and to maternal care, as well as increased labour force participation (Avula et al., 2022). Tamil Nadu was singled out for particular improvements in women’s status and care, linked to a strong legacy of policies such as improved sex ratio, girls’ education, and raising the age at marriage, alongside health initiatives.

Only one of the studies, in Vietnam, delves deeper, as a specific objective, to consider equity in outcomes and access to relevant government services (Harris et al., 2021). The study finds significant differences in stunting outcomes between the majority ethnic Kinh population and minority ethnicities. Many of Vietnam’s 54 recognised ethnic groups living below the poverty line do not benefit from Vietnam’s significant economic growth and improvement in living standards over the past couple of decades – particularly smaller ethnic groups in the central and northern highland regions. Uniquely, the study charted differences between majority and minority ethnicities in the underlying determinants of malnutrition, including measures of wealth and women’s educational attainment and coverage of key health interventions such as immunization, birth, and antenatal care. Consistently large gaps were seen in all these indicators between majority and minority ethnic groups. Stakeholder interviews, media analysis and policy review also pointed to the need to better take into account the needs and preferences of minority ethnicities, rather than hope for their assimilation in mainstream politics and national growth. Additionally, in Ghana and Nigeria, there were inequalities in stunting across regions, with southern regions having much lower stunting rates compared to northern regions. To a lesser extent, similar inequalities were reported for anaemia in Ghana (Aryeetey et al., 2021). A projected analysis of future stunting prevalence in Nigeria found that closing 2018 inequities in intervention coverage across regions (referred to as geopolitical zones) had the potential to reduce national stunting by 26%, compared to an 8% reduction if intervention coverage improved according to existing trends (Adeyemi et al., 2022).

In the relatively high-income context of Brighton in the UK, poverty is strongly correlated with increased childhood obesity (Salm et al., Forthcoming). In poorer communities, food environments and infrastructure inhibit access to a healthy diet. Despite an active network of services and initiatives to target health and obesity inequalities, these communities continue to have much higher rates of overweight and obesity. There is consensus across stakeholders interviewed that without targeting upstream social determinants (of adequate income, education, housing) local action will not be sufficient to change the nutrition outcomes of these communities.

### Commitment, coherence and capacity

A specific aim of this, and earlier waves of Stories of Change in Nutrition studies, has been to analyse whether policy environments in the study countries form an *enabling environment* i.e. ‘‘political and policy processes that build and sustain momentum for the effective implementation of actions that reduce malnutrition’’ (Gillespie et al., 2013). Many of the studies undertake this as part of the triangulation between different methods, although some studies are focused only on the policy environment (Drimie et al., Forthcoming). The studies use different policy analysis frameworks to help accomplish this aim – many opting to build on the 2013 Lancet series’ characterisation of an enabling environment (Gillespie et al., 2013), while others adapt political, policy science, or health policy frameworks to focus on specific questions such the role of advocacy coalitions in policy processes (Harris et al., 2022), or the status of implementation. We partly follow the 2017 synthesis of *Stories of Change* studies (Gillespie & van den Bold, 2017) in using a simplified framework which focuses on key areas of *policy and political commitment* to nutrition, *coherence* across sectors (including issues of vertical (implementation) and horizontal (multi-sectoral) co-ordination); and *capacity*.^3^ While there are many different ways of studying nutrition policy environments (Baker et al., 2018; Gillespie et al., 2013; te Lintelo & Lakshman, 2015), these sub-themes consistently remain key concerns of stakeholders in many of the countries studied.

In terms of *policy and political commitment*, many of the qualitative public policy studies report favourable changes in policy environments over their study periods. Across policy document analysis we see an increasingly sophisticated array of policies under development with relevance to nutrition objectives. Mapping actor networks, which took place in Ghana, Nigeria, and Burkina Faso, reveal varying degrees of connectivity between different nutrition actors and a large number of active participants in these networks, suggesting a well-established sector. Ministries of Health were most frequently identified as central nodes, alongside other government actors, except in Nigeria where UNICEF was a central node. The centrality of development partners (bilateral and multilateral donors and other actors such as BMGF and civil society) differed from country to country. While the resources and technical assistance provided by such actors was highlighted as positive, in a few countries (e.g., Burkina Faso, Ghana, Nigeria) the donor-dependency in terms of resources, ownership and ideas, was highlighted as a challenge and vulnerability, particularly if external funding were to be cut. In many countries, particularly Ghana, India, Burkina Faso and Nigeria, civil society contributed a key role in advocating for effective nutrition policy and political commitment, as well as working variably on technical support and programme delivery (Adeyemi et al., 2022; Aryeetey et al., 2021; Avula et al., 2022; Turowska et al., Forthcoming). The role of the Scaling Up Nutrition (SUN) movement was singled out as having helped drive policy commitment in Ghana, Nigeria, and Burkina Faso, as were international events such as the intergovernmental International Conference on Nutrition in 2014. In some countries, such as Burkina Faso, a strong divide still exists between different actor coalitions working on agriculture, broader issues of food security or hunger; and coalitions working on malnutrition. Actor mapping highlights these distinct coalitions, but with some actors placed in both, suggesting opportunities for greater coherence in future for food and nutrition security; though this remains a challenge (Turowska et al., Forthcoming).

In terms of *coherence*, issues of co-ordination between sectors and effective co-location of sectoral programs in communities themselves was a theme across many of the studies. These included positive progress (in Burkina Faso and Rwanda (Iruhiriye et al., 2022; Turowska et al., Forthcoming)) as well as ongoing challenges, e.g. where commitment is only rhetorical (Baker et al., 2018) – an example being the National Food and Nutrition Security Council in South Africa which is promised but not yet established as an active body (Drimie et al., Forthcoming). What counts as evidence of effective multisectoral co-ordination was also interpreted differently by stakeholders across the different study countries. In some countries, the existence of a co-ordinating body at a national level was seen as good evidence of the commitment to policy coherence across sectors. This was accompanied by a general acceptance amongst stakeholders that a multi-sectoral approach was a necessary step for tackling different forms of malnutrition. In Burkina Faso, the creation of a new co-ordination body under the powers of the President was seen as a potential step in overcoming dissonance between food security and nutrition communities, despite these same challenges remaining for the new body to overcome (Turowska et al., Forthcoming). In Rwanda, the Food and Nutrition Secretariat, located within the Ministry of Local Government, was viewed positively by stakeholders for its co-ordinating role^4^ (Iruhiriye et al., 2022). In Ghana, stakeholders felt more co-ordination was needed and so they called for the creation of an empowered national nutrition commission to help overcome the challenges besetting sectors in engaging with the National Development Planning Commission, the central actor for implementation (Aryeetey et al., 2021). In Nigeria, the inauguration of a National Council on Nutrition and a growing capacity to coordinate and harmonise implementation at a technical level were seen as positives; but overall, actor mapping revealed a high level of fragmentation and many cliques, with capacity for multisectoral co-ordination remaining weak (Adeyemi et al., Forthcoming). In India, two national level programmes: the National Health Mission (NHM; formerly the National Rural Health Mission) and the Integrated Child Development Services (ICDS) have driven a policy focus towards a community level approach (Avula et al., 2022). In the case of Brighton, having a Healthy Weight Programme Board was seen as key to bring together a diverse range of actors (such as those working in city planning, transport, the private sector, public health etc.) and establish a common strategy around healthy weight in the city (Salm et al., Forthcoming). This allowed for networks and initiatives to form around a shared agenda, thus improving efficiency and reducing duplication of services.

While many countries noted improved political will and a national level co-ordination and policy landscape, the policy and programmatic landscape for the actual delivery of interventions was consistently highlighted as a challenge in Burkina Faso, Ghana and Nigeria (Adeyemi et al., 2022; Adeyemi et al., Forthcoming; Aryeetey et al., 2021). Reasons listed by stakeholders included limited funding, inadequate coverage and quality of interventions, suboptimal human resources and other capacity and security issues. In South Africa, the policy environment is quite robust, but the implementation of the policies into appropriate coordination frameworks and implementation mechanisms at provincial and local level remains a big challenge, although the Western Cape was highlighted as having made significant progress on this (Drimie et al., Forthcoming).

Linkages between cross sectoral ‘horizontal’ co-ordination and national-to-local ‘vertical’ delivery were examined in all studies that focused on the sub-national level: Rwanda, Nigeria, South Africa, Brighton (the UK) and India. In Rwanda, for example, an increased level of decentralization has been accompanied by subnational and national level co-ordination platforms (Iruhiriye et al., 2022). Mid-level actors have been granted a greater share of powers and have gained an increased ability to plan, implement and monitor nutrition programmes. Integrating co-ordination platforms within existing political and administrative structures helped overcome the lack of political will to take forward sub-national work seen in other studies of decentralized co-ordination (Hoey & Pelletier, 2011). In Jigawa and Kaduna states in Nigeria, increased advocacy, multisectoral actions and coordination structures for nutrition, domestication of national policies, development of state and local level operational plans, and budgetary allocations to nutrition at the state and local government level were seen by stakeholders as indications of positive sub-national nutrition policy environment changes (Adeyemi et al., 2022). In India, similarly, the ability of states, state-level politicians and bureaucrats to modify, ‘top-up’ and otherwise innovate around national level programmes such as the ICDS or NRHM) (now National Health Mission – NHM), was seen as helping to explain the relative success – compared to national level progress – of the four states studied (Avula et al., 2022). State level champions were important in Odisha, India, and Nigeria for increasing political commitment and turning commitment into programmatic changes. In South Africa, interviewees highlighted that despite coordinated planning between sectors at national level, there was a lack of integrated structures at district levels leading to siloed implementation. There was however some evidence of effective structures at lower administrative levels in e.g. Kwazulu-Natal and Western Cape (Drimie et al., Forthcoming). Finally, in India, studies highlighted how political stability in all four states was crucial for implementing health and nutrition interventions. In Odisha for example, the same political party was in power for three terms, providing tenure stability for bureaucrats working on nutrition and health programs (Avula et al., 2022).

In terms of *capacity*, several studies noted an improved enabling environment around the use of research and evidence more generally. Mistrust of some forms of survey data was reported in Nigeria and data quality and quantity is still perceived as inadequate, though improving (Adeyemi et al., 2022). Generally, increasing data availability at sub-national and national levels was seen as important for the planning process at national and sub-national levels (Adeyemi et al., 2022; Avula et al., 2022; Turowska et al., Forthcoming). Data gaps still remain, including for outcomes for important groups such as adolescents, or on anaemia, or on wider indicators of food security not covered by DHS. Only the study in Vietnam reports on data disaggregated by ethnicity to assess equity amongst sub-groups (see below) (Harris et al., 2021).

State investment in the community workforce was a particularly important part of the increase in capacity in some countries. In India, for example the ICDS was expanded between 2001 and 2007 and the NHM launched in 2005–06, both providing significant increases in resources and health worker capacity at a community level. The NHM now accounts for nearly half of the national level (Union) health budget and has increased the focus on primary care (Avula et al., 2022). Similar significant improvements in nutrition or broader health sector capacity were reported in Ghana (Aryeetey et al., 2021). In Burkina Faso, although community members report an increase in human resources at local health centres, national-level stakeholders emphasized that lack of vertical coordination posed challenges in organizing human resources for nutrition at decentralized levels (Becquey et al., 2022).

## Discussion

These Stories of Change studies provide a unique insight into the determinants of nutritional change in 14 studies across 9 countries; and build on a previous wave of studies (Gillespie et al., 2017). A summary of the earlier generation of studies concluded that: “health care, household wealth, and parental education are important predictors of stunting declines in most countries. and that “roles of other determinants vary across [the 19] case studies, emphasising the importance of context “ (Heidkamp et al., 2021, pp. 1407–1408).

The current generation of studies both complement and deepen these findings. These studies are essential accompaniments to routine measurement of change undertaken via regular Demographic and Health Surveys, Multiple Indicator Cluster Surveys, or routine health Management Information Systems. While such surveys provide essential data on outcomes and, to some extent, programme coverage, they provide little further analysis of why change happened, how and whether policy targets are being met (Adeyemi et al., 2022), nor certain disaggregated impacts on particular sub-national regions or population groups.

The studies in this volume took a deliberately multi-sectoral approach, looking for evidence of different sectoral contributions to improvements in nutrition. Consistent with earlier studies, improvements are not driven by one sector, but come from health, education, sanitation as well as ‘nutrition specific’ measures. All these sectoral contributions are in turn based on a bedrock of pro-poor development: the distribution of wealth and opportunities to households (as measured by the asset index and parental levels of education) which was important across all studies that examined DHS data. Maternal education was important in most studies, as was maternal health support such as access to antenatal care. Such investments in broad-based people-centred development requires as much political will as that needed to invest in nutrition-specific interventions. If population wide improvements are to benefit specific groups, such as the ethnic minorities studied in Vietnam or those subject to the racial disparities highlighted in South Africa, then tackling entrenched histories of discrimination in distribution of wealth and access to government services will also need to be addressed. The long-term challenges in South Africa are to narrow racial disparities in wealth, health, and education and to generate opportunities for many more people, especially those historically disadvantaged. This is essential for more people to lead healthy, productive lives. Further, in South Africa and elsewhere, it is critical for the nutrition profession to become more racially diverse, as it is currently a space where advantaged white people often dominate. Decolonising practice therefore holds potential for advancing nutrition across the board.

Driving nutritional change are the key actors and institutions examined via policy process analysis in the majority of studies. Here studies help move beyond the jargon of international policy recommendations that nutrition should be multisectoral, or locally owned: many of the studies provide helpful day to day examples of the challenges of coordination at national and local, decentralised levels. Beyond the rhetoric, understanding the views and incentives of those working within the multiple different sectors that are relevant to nutritional change is no easy feat. The small city of Brighton and Hove on the UK’s south coast provides one example where a ‘whole system’ approach to a nutritional problem – child obesity– has been embraced wholeheartedly, bringing in stakeholders from multiple sectors and different types of local organisations. In Rwanda, functioning district level co-ordination was also very much a reality, enhanced by the leadership of mid-level actors. On a national scale, multisectoral co-ordination bodies are in operation in many of the countries studied (such as Ghana, Burkina Faso, Rwanda, and Nigeria), and are seen as important in bringing key stakeholders, particularly government, around the same table. These national processes are experiencing variable degrees of success; evidence of effective translation of multisectoral contributions into reality via implementation and monitoring of plans is thin on the ground. ‘Face-to-face’ multisectoralism (i.e. better integrated services at the point of delivery) may work well when implemented at local levels, also providing the possibility of stronger engagement with communities; whereas national levels need to coordinate at the level of policy development, funding, monitoring/evaluation, and accountability mechanisms. In Burkina Faso, such co-location at the local level of core interventions from multiple sectors may also have led to positive outcomes.

Despite the continued drive towards multisectoralism, however, one study in this volume (Avula et al., 2022) highlights how nutrition advocates may need to play a different role in the future than simply expecting every sector to adopt nutrition targets: “Too often, nutrition strategies ask that other sectors integrate nutrition into their programs and actions; however, our findings, together with those from other countries, highlight that core sectoral actions to address known determinants of poor child growth are important drivers of change.”(Avula et al., 2022). These findings are echoed across many of the studies, and with newer nutrition challenges on the horizon for many of the countries, international nutrition advocates need to assess how to be a strategic ally to those working in other sectors as a means of accomplishing mutual goals, rather than expecting every sector to be ‘nutrition sensitive’.

Much of the change – or lack of change – seen across the studies can also be assessed in terms of power: The relative power (or disempowerment) of different population groups to access the determinants of good nutrition; the power to set policy agendas or have strategic food system goals realised; or the power to participate or have voice in social and political decisions related to nutrition. The key role of power in social and political contexts has been recognised in previous nutrition research (Harris, 2019; Nisbett et al., 2014; Walls et al., 2020) and is illustrated implicitly and explicitly across this body of work. For nutrition allies and advocates, attention to the role of power in food systems, policy systems and social systems will be important in understanding and creating the changes suggested in this synthesis.

## Conclusions

These 14 studies of nutritional change complement an earlier series (Gillespie & van den Bold, 2017) and other similar exercises^5^ in providing country level or sub-national, multifaceted, multi-methods ‘stories of change’ in nutritional outcomes. Here the series extends beyond the original focus on child stunting and wasting to other outcomes including anaemia and obesity and overweight. In many countries, similar determinants, including better distribution of wealth, education, maternal and child health and nutrition services, or wider community factors including sanitation have been associated with improvements in nutritional status. Given this inherent multisectorality – and the relative immaturity of the policy sector in dealing with new challenges such as obesity and overweight and associated influences in the wider food environment – many countries are experimenting with different models of ensuring coherence across sectors, whilst simultaneously tackling nutrition’s upstream (social/economic) and downstream (health and dietary) determinants. Although this is a huge challenge, progress is generally positive on outcomes such as stunting and anaemia, with some global ‘islands of innovation’ to draw on in terms of whole-systems approaches to obesity and overweight. All the studies provide wider lessons that will be valuable to researchers and policy makers across the study countries and elsewhere.

## Footnotes

1 There are 14 studies covered here over 9 countries: 4 studies took place in India; and 3 separate studies in Vietnam.

2 In most studies the asset index was associated with the greatest reduction in outcomes of interest, but in Nigeria, maternal education was associated with the largest reduction, followed by the asset index. In Rwanda, health-related services (not household asset score) were the largest drivers of change.

3 The 2017 series focused on commitment, coherence and community, but given that many of the articles in this series do not report on community or local government findings (but have done so or will do elsewhere,), we do not summarise community level findings here.

4 Subsequent to the study, the Secretariat became the National Child Development Agency, located in the Ministry of Gender and Family Promotion.

5 https://www.exemplars.health/topics/stunting/cross-country-synthesis last accessed 20/12/2021.
